# Supplementary figures and images for: Additive effects of Trichoderma isolates for enhancing growth, suppressing southern blight and modulating plant defense enzymes in tomato
Source: PLoS One. 2025 Jul 30;20(7):e0329368. doi: 10.1371/journal.pone.0329368 (PMC12310031; doi:10.1371/journal.pone.0329368)

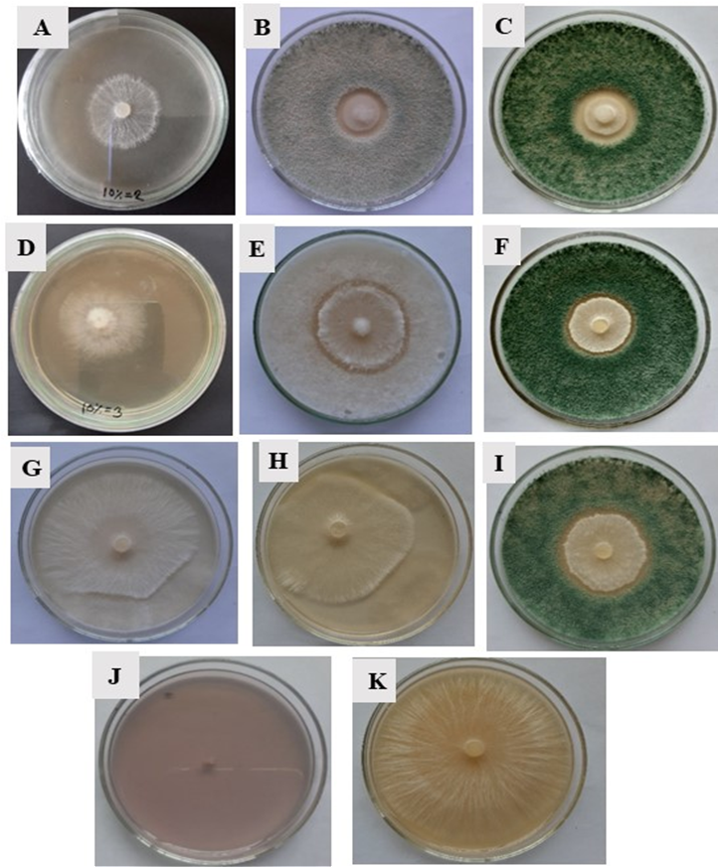

Supplement: S1 Fig — Panels show the following treatments: Tri2 (A – 10%, B – 20%, C – 30%), Tri3 (D – 10%, E – 20%, F – 30%), Tri6 (G – 10%, H – 20%, I – 30%), J – Provax-200 at 200 ppm, and K – S. rolfsii (control). (TIF) [file pone.0329368.s001.tif]

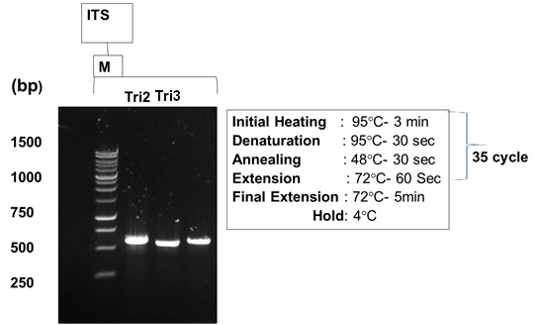

Supplement: S2 Fig — M: 1 kb DNA ladder (marker). (TIF) [file pone.0329368.s002.tif]

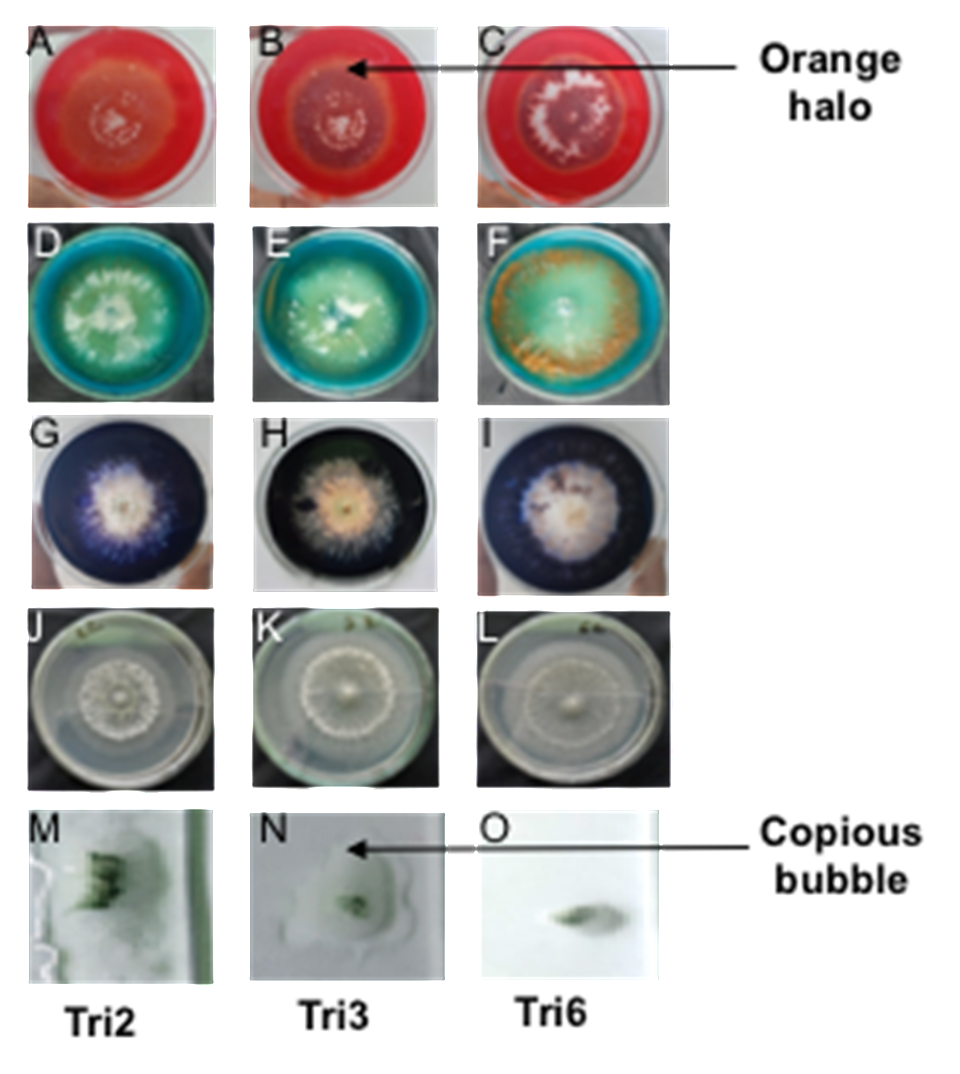

Supplement: S3 Fig — Panels show the following enzyme reactions: cellulose (A – C), protease (D- F), amylase (G – I), lipase (J – L), and catalase (M – O). Tri2, Tri3, and Tri6 represent different Trichoderma isolates. (TIF) [file pone.0329368.s003.tif]

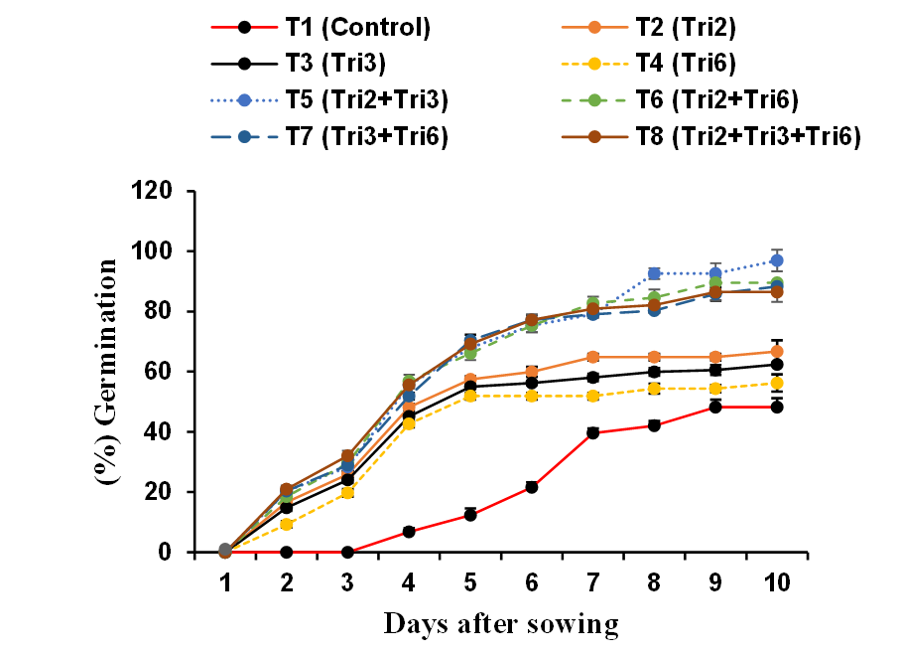

Supplement: S4 Fig — Tri2, Tri3, and Tri6 represent different Trichoderma isolates. A Fisher’s LSD test (p < 0.05) was performed to identify significant differences among the treatments. (TIF) [file pone.0329368.s004.tif]

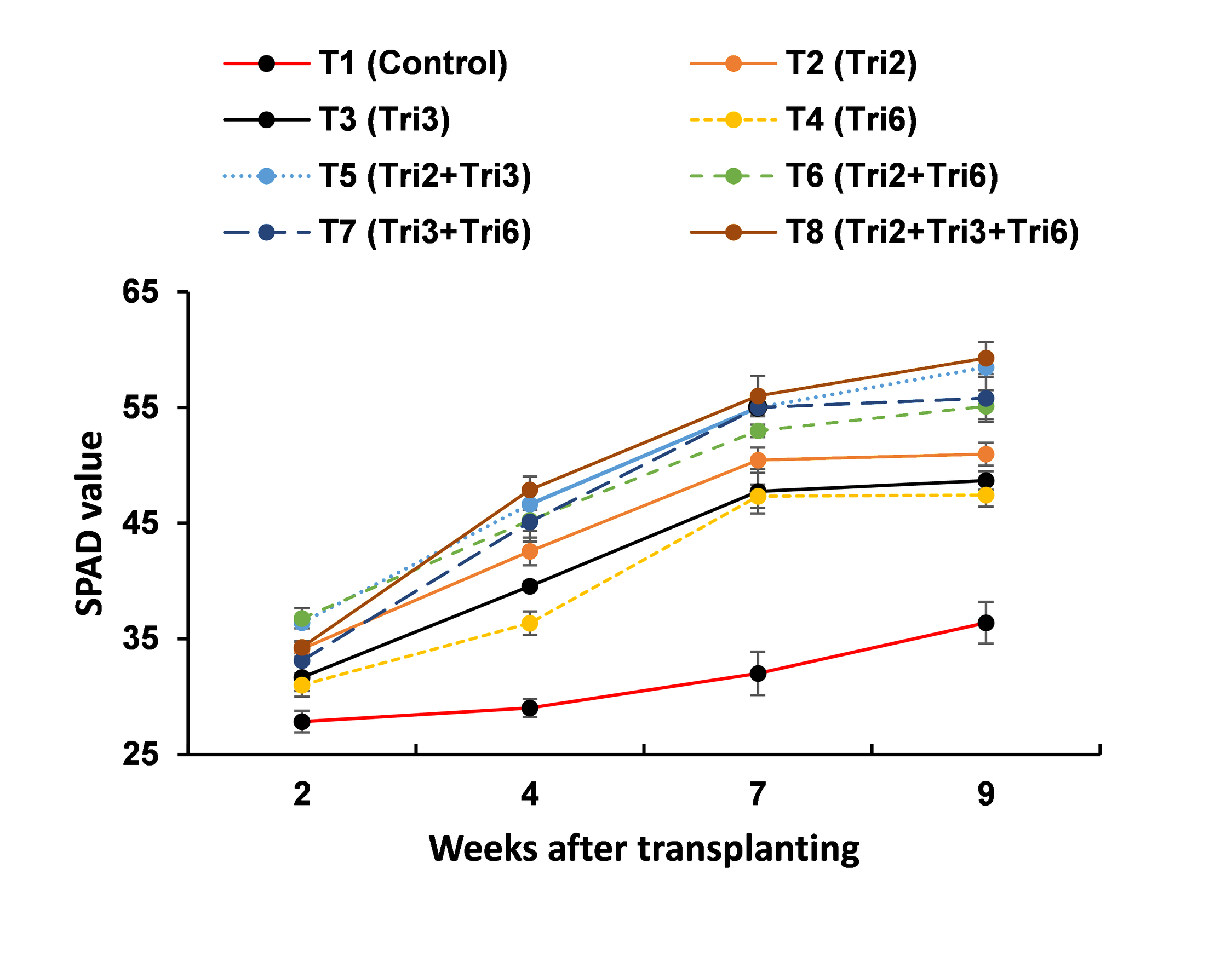

Supplement: S5 Fig — SPAD readings were recorded at 4, 5, 7, and 9 weeks after transplanting. In the control treatment (T1), plants were not inoculated with any Trichoderma isolates. For the other treatments, Tri2, Tri3, and Tri6 represent plants treated with Trichoderma isolates Tri2, Tri3, and Tri6, respectively. The data are presented as mean ± SE, with values derived from three biological replicates (n = 3) for each treatment. (TIF) [file pone.0329368.s005.tif]

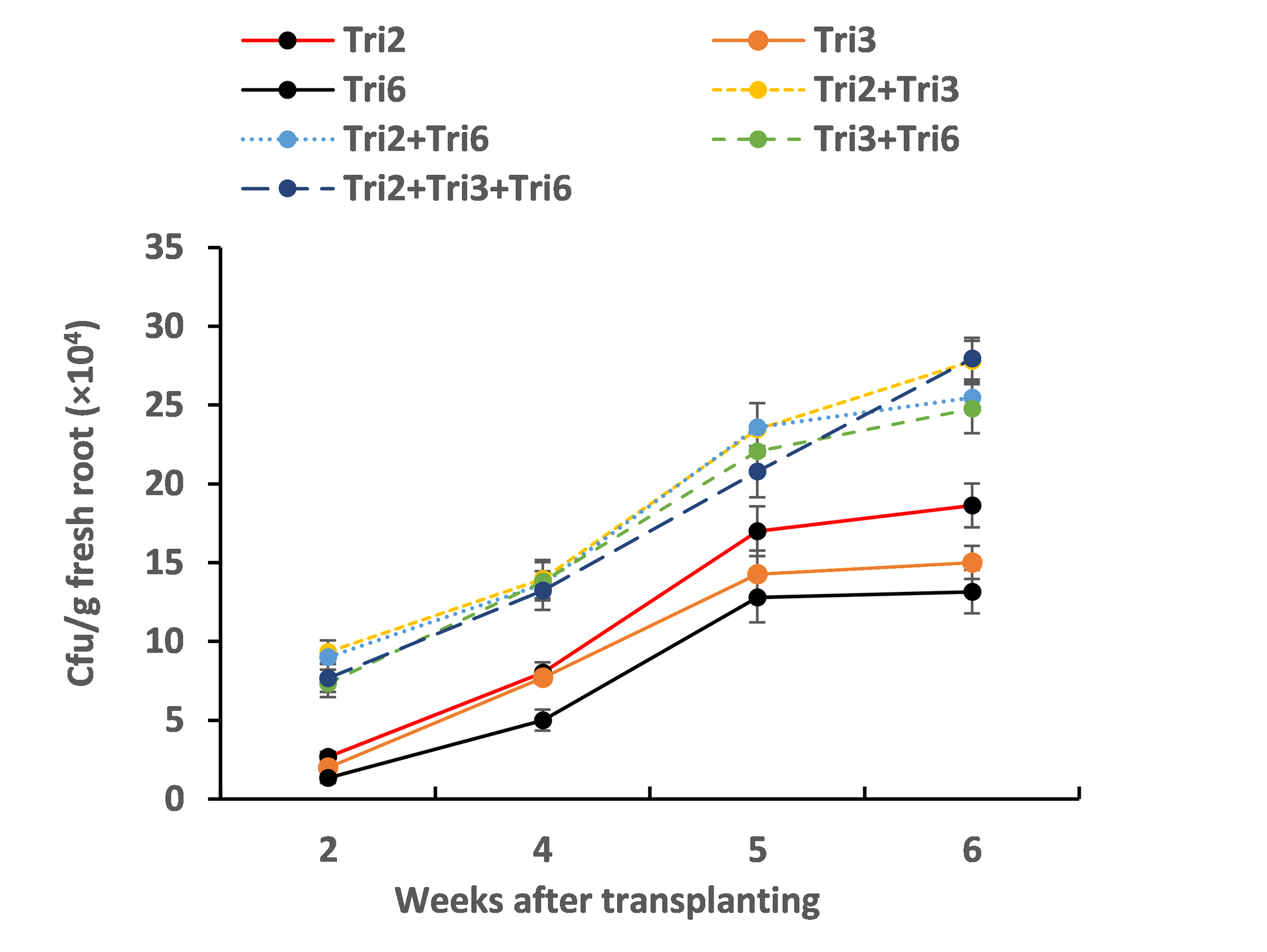

Supplement: S6 Fig — The data are presented as the number of colony-forming units (CFU) per gram of fresh root weight (mean ± standard error). Root samples were collected from three sets of four plants at each time point (2, 4, 5, and 6 weeks post-transplant). In the treatments, Tri2, Tri3, and Tri6 represent different Trichoderma isolates. (TIF) [file pone.0329368.s006.tif]

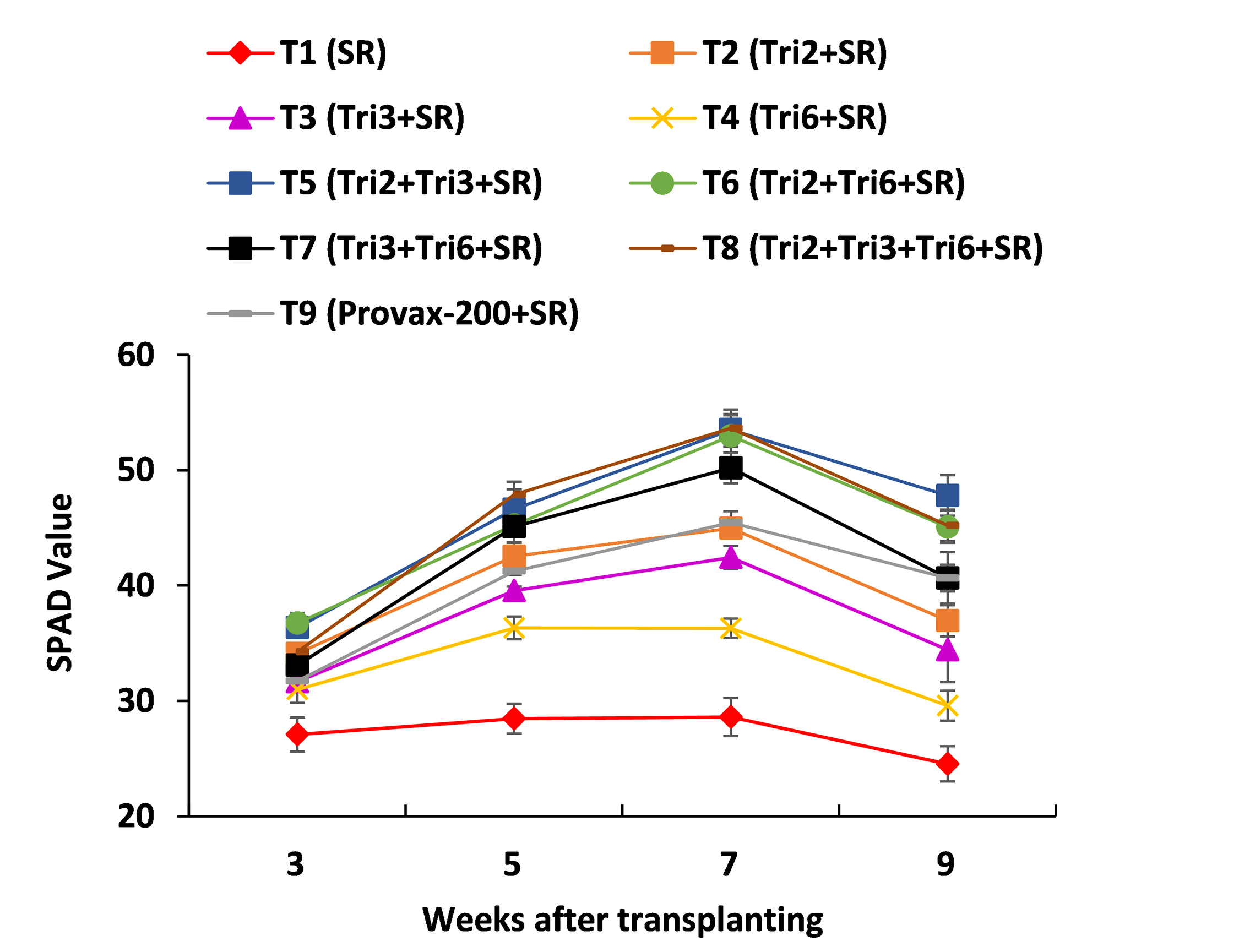

Supplement: S7 Fig — SPAD readings were recorded for 4, 5, 7, and 9-week-old transplanted tomato plants across various treatments. In the treatments, “SR” indicates inoculation with the Southern blight pathogen Sclerotium rolfsii, while Tri2, Tri3, and Tri6 represent treatments with Trichoderma isolates Tri2, Tri3, and Tri6, respectively. Treatment T9 includes the application of the fungicide Provax-200. Data are presented as mean ± SE, with values obtained from three biological replicates (n = 3) for each treatment. (TIF) [file pone.0329368.s007.tif]
