# Supplementary material for: Additive effects of Trichoderma isolates for enhancing growth, suppressing southern blight and modulating plant defense enzymes in tomato
Source: PLoS One. 2025 Jul 30;20(7):e0329368. doi: 10.1371/journal.pone.0329368 (PMC12310031; doi:10.1371/journal.pone.0329368)
Supplement: S1 Table — (DOCX) [file pone.0329368.s008.docx]

**S1 Table. List of *Trichoderma* isolates, host plants and locations of collections.**

| ***Trichoderma* isolates** | **Rhizosphe Crop** | **Locations** |
| --- | --- | --- |
| **Tri1** | Tomato (*Solanum lycopersicum*) | Research field, BSMRAU, Gazipur, Bangladesh |
| **Tri2** | Soybean (*Glycine max*) | Subarnachar, Noakhali, Bangladesh. |
| **Tri3** | Soybean (*Glycine max*) | Subarnachar, Noakhali, Bangladesh |
| **Tri4** | Peanut (*Arachis hypogaea*) | Char Alexander, Lakshmipur, Bangladesh |
| **Tri5** | Eggplant (*Solanum melongena*) | Ramgati, Lakshmipur, Bangladesh |
| **Tri6** | Cucumber (*Cucumis sativus*) | Subarnachar, Noakhali, Bangladesh |
| **Tri7** | Peanut (*Arachis hypogaea*) | Ramgati, Lakshmipur Bangladesh |
| **Tri8** | Soybean (*Glycine max*) | Sonapur, Noakhali, Bangladesh |
| **Tri9** | Tomato (*Solanum lycopersicum*) | Haimchar, Chandpur, Bangladesh |
| **Tri10** | Eggplant (*Solanum melongena*) | Research field, BSMRAU, Gazipur, Bangladesh. |
