# Supplementary material for: Additive effects of Trichoderma isolates for enhancing growth, suppressing southern blight and modulating plant defense enzymes in tomato
Source: PLoS One. 2025 Jul 30;20(7):e0329368. doi: 10.1371/journal.pone.0329368 (PMC12310031; doi:10.1371/journal.pone.0329368)
Supplement: S2 Table — (DOCX) [file pone.0329368.s009.docx]

**S2 Table.** **Sequencing homology test of the ITS region of the selected *Trichoderma* isolates with significant alignments using BLAST software.**

| **Fungus isolates** | **NCBI Accession** | **Reference Species** | **Percent Identity** |
| --- | --- | --- | --- |
| **Tri2** | OR678071 | *Trichoderma asperellum* | 99.68% |
| **Tri3** | OR678072 | *Trichoderma asperellum* | 99.53% |
| **Tri6** | OR678073 | *Trichoderma asperellum* | 99.68% |
