# Supplementary material for: Additive effects of Trichoderma isolates for enhancing growth, suppressing southern blight and modulating plant defense enzymes in tomato
Source: PLoS One. 2025 Jul 30;20(7):e0329368. doi: 10.1371/journal.pone.0329368 (PMC12310031; doi:10.1371/journal.pone.0329368)
Supplement: S3 Table — (+) Isolates showing weak enzyme activity, (++) Isolates showing moderate enzyme activity and (+++) Isolates showing strong enzyme activity. (DOCX) [file pone.0329368.s010.docx]

**S3 Table.** **Plant growth-promoting and biocontrol traits of selected *Trichoderma* isolates.**

| **Characterization test** | | ***Trichoderma* isolates** | | |
| --- | --- | --- | --- | --- |
|  |  | **Tri2** | **Tri3** | **Tri6** |
| **Growth-promoting traits** | Phosphate solubilization | +++* | +++ | ++ |
| **Bio-control traits** | Cellulase assay | +++ | +++ | ++ |
|  | Protease assay | +++ | +++ | ++ |
|  | Amylase assay | +++ | +++ | ++ |
|  | Lipase assay | +++ | +++ | ++ |
|  | Catalase test | +++ | +++ | ++ |

**Note*: (+) Isolates showing weak enzyme activity, (++) Isolates showing moderate enzyme activity and (+++) Isolates showing strong enzyme activity
