# Supplementary material for: Additive effects of Trichoderma isolates for enhancing growth, suppressing southern blight and modulating plant defense enzymes in tomato
Source: PLoS One. 2025 Jul 30;20(7):e0329368. doi: 10.1371/journal.pone.0329368 (PMC12310031; doi:10.1371/journal.pone.0329368)
Supplement: S4 Table — Values (mean ± SE) for each treatment were obtained from three biological replicates (n = 3). Different letters within each column indicate significant differences, as determined by Fisher’s LSD test (p < 0.05). Values in parentheses represent the percentage increase relative to the control. (DOCX) [file pone.0329368.s011.docx]

**S4 Table. Effect of selected *Trichoderma* isolates on the tomato seed germination, seedling height and seedling vigor in *in vitro* assays.**

| **Treatment** | **Germination (%)** | **Seedling height (cm)** | **Seedling vigor** |
| --- | --- | --- | --- |
| **Control** | 71.00±0.80d | 4.00±0.89b | 283.67±0.89b |
| ***Trichoderma* isolate Tri2** | 94.67±0.96a*  (33.34)** | 8.00±0.91a  (100) | 756.67±10.56a  (166.74) |
| ***Trichoderma* isolate Tri3** | 89.00±1.53b  (25.35) | 7.00±1.42a  (75) | 622.33±7.98a  (119.38) |
| ***Trichoderma* isolate Tri6** | 80.33±2.56c  (13.14) | 5.00±2.43b  (50) | 402.00±5.38b  (41.71) |

**Note*: Values (mean ± SE) for each treatment were obtained from three biological replicates (*n = 3*). Different letters within each column indicate significant differences, as determined by Fisher’s LSD test (*p < 0.05*). Values in parentheses represent the percentage increase relative to the control.
