# Supplementary material for: Additive effects of Trichoderma isolates for enhancing growth, suppressing southern blight and modulating plant defense enzymes in tomato
Source: PLoS One. 2025 Jul 30;20(7):e0329368. doi: 10.1371/journal.pone.0329368 (PMC12310031; doi:10.1371/journal.pone.0329368)
Supplement: S5 Table — In treatments, Tri2, Tri3, and Tri6 represent treatments with Trichoderma isolates Tri2, Tri3, and Tri6, respectively. Values (mean ± SE) for each treatment were obtained from three biological replicates (n = 3). Different letters within each column indicate significant differences, as determined by Fisher’s LSD test (p < 0.05). Values in parentheses represent the percentage increase relative to the control. (DOCX) [file pone.0329368.s012.docx]

**S5 Table.** **Effect of single, dual and triple combinations of *Trichoderma* treatments on photosynthetic pigments in 21-day seedlings of tomato.**

| **Treatment** | **Chlorophyll *a* (mg g^−1^ FW)** | **chlorophyll *b* (mg g^−1^ FW)** | **Total chlorophyll (mg g^−1^ FW)** | **Carotenoids (mg g^−1^ FW)** |
| --- | --- | --- | --- | --- |
| **T1 (Control)** | 0.51±0.31b | 0.04±0.24d | 0.52±0.10d | 0.23±0.10c |
| **T2 (Tri2)** | 0.54±0.01b  (10.03) | 0.37±0.11bc  (866.75) | 0.92±0.10c  (77.56) | 0.25±0.10c  (10.03) |
| **T3 (Tri3)** | 0.54±0.10b  (7.16) | 0.36±0.10c  (800.75) | 0.90±0.09c  (72.44) | 0.25±0.03c  (7.16) |
| **T4 (Tri6)** | 0.54±0.10b  (7.16) | 0.36±0.09c  (800.00) | 0.89±0.10c  (72.44) | 0.25±0.03c  (7.16) |
| **T5 (Tri2+Tri3)** | 1.38±0.05a  (222.89) | 0.82±0.10a  (1950.00) | 2.20±0.10a  (322.44) | 0.75±0.04a  (222.89) |
| **T6 (Tri2+Tri6)** | 1.42±0.05a  (205.74) | 0.63±0.02ab  (1483.25) | 2.05±0.02ab  (294.23) | 0.71±0.03a  (205.74) |
| **T7 (Tri3+Tri6)** | 1.30±0.04a  (141.45) | 0.60±0.05abc  (1416.75) | 1.91±0.07b  (267.94) | 0.56±0.06b  (141.45) |
| **T8 (Tri2+Tri3+Tri6)** | 1.38±0.07a  (155.77) | 0.65±0.03a  (1541.75) | 1.94±0.07b  (273.08) | 0.59±0.02b  (155.77) |

*Note*: In treatments, Tri2, Tri3, and Tri6 represent treatments with *Trichoderma* isolates Tri2, Tri3, and Tri6, respectively. Values (mean ± SE) for each treatment were obtained from three biological replicates (*n = 3*). Different letters within each column indicate significant differences, as determined by Fisher’s LSD test (*p < 0.05*). Values in parentheses represent the percentage increase relative to the control.
