# Supplementary material for: Additive effects of Trichoderma isolates for enhancing growth, suppressing southern blight and modulating plant defense enzymes in tomato
Source: PLoS One. 2025 Jul 30;20(7):e0329368. doi: 10.1371/journal.pone.0329368 (PMC12310031; doi:10.1371/journal.pone.0329368)
Supplement: S6 Table — In treatments, Tri2, Tri3, and Tri6 represent treatments with Trichoderma isolates Tri2, Tri3, and Tri6, respectively. Values (mean ± SE) for each treatment were obtained from three biological replicates (n = 3). Different letters within each column indicate significant differences, as determined by Fisher’s LSD test (p < 0.05). Values in parentheses represent the percentage increase relative to the control. (DOCX) [file pone.0329368.s013.docx]

**S6 Table.** **Gas exchange attributes of tomato foliage in plants treated with single, dual, and triple combinations of *Trichoderma* isolates.**

| **Treatment** | **Photosynthetic rate (µmolco_2_m­²s­¹)** | **Leaf temperature (°C)** | **Transpiration rate (nmolH_2_Om-²s-¹)** | **Stomata conductance to H_2_O (molH_2_Om-²s-¹)** |
| --- | --- | --- | --- | --- |
| **T1 (Control)** | 4.87 ± 1.14f | 1.75 ± 0.14e | 0.93 ± 0.01a | 0.32 ± 0.01a |
| **T2 (Tri2)** | 12.01±0.26d  (167.28) | 3.50± 0.47d  (99.81) | 0.38 ± 0.01c  (-59.43) | 0.19± 0.03b  (-40.06) |
| **T3 (Tri3)** | 11.80± 0.27d  (142.44) | 2.58± 0.21de  (47.34) | 0.41 ± 0.04c  (-55.87) | 0.18 ± 0.03b  (-42.12) |
| **T4 (Tri6)** | 8.10± 1.42e  (66.39) | 2.46± 0.15de  (40.68) | 0.52 ± 0.04b  (-44.48) | 0.17 ± 0.01b  (-46.32) |
| **T5 (Tri2+Tri3)** | 19.85± 0.42a  (307.74) | 8.59± 0.82a  (390.32) | 0.17 ± 0.04d  (-81.49) | 0.04 ± 0.01c  (-86.51) |
| **T6 (Tri2+Tri6)** | 15.05 ± 0.39bc  (209.03) | 5.94±0.32bc  (239.17 | 0.23 ± 0.02d  (-75.09) | 0.07 ± 0.01c  (-77.58) |
| **T7 (Tri3+Tri6)** | 13.93± 0.60bcd  (185.68) | 5.40± 0.35c  (207.99) | 0.23 ± 0.02d  (-74.73) | 0.10± 0.00c  (-76.85) |
| **T8 (Tri2+Tri3+Tri6)** | 16.38± 1.25b  (234.48) | 6.74± 0.21b  (284.80) | 0.22 ± 0.01d  (-75.79) | 0.09 ± 0.00c  (-69.46) |

*Note*: In treatments, Tri2, Tri3, and Tri6 represent treatments with *Trichoderma* isolates Tri2, Tri3, and Tri6, respectively. Values (mean ± SE) for each treatment were obtained from three biological replicates (*n = 3*). Different letters within each column indicate significant differences, as determined by Fisher’s LSD test (*p < 0.05*). Values in parentheses represent the percentage increase relative to the control.
