# Supplementary material for: Additive effects of Trichoderma isolates for enhancing growth, suppressing southern blight and modulating plant defense enzymes in tomato
Source: PLoS One. 2025 Jul 30;20(7):e0329368. doi: 10.1371/journal.pone.0329368 (PMC12310031; doi:10.1371/journal.pone.0329368)
Supplement: S8 Table — In the treatments, SR denotes inoculation with the Southern blight pathogen Sclerotium rolfsii, while Tri2, Tri3, and Tri6 represent treatments with Trichoderma isolates Tri2, Tri3, and Tri6, respectively. In T9, treatment with the fungicide Provax-200 was included. Values (mean ± SE) for each treatment were obtained from three biological replicates (n = 3). Different letters within each column indicate significant differences, as determined by Fisher’s LSD test (p < 0.05). (DOCX) [file pone.0329368.s015.docx]

**S8 Table. Effect of application of single, dual, and triple combinations of *Trichoderma* on the production of Sclerotia on the soil surface by *Sclerotium rolfsii* in seed trays at different weeks after sowing (WAS).**

| **Treatment** | **Number of sclerotia/cm^2^** | | | |
| --- | --- | --- | --- | --- |
|  | **1 WAS** | **2 WAS** | **3 WAS** | **4 WAS** |
| T1 (SR) | 5.33 ± 2.03a | 11.91 ± 0.96a | 19.00 ± 2.65a | 21.00 ± 1.53a |
| T2 (Tri2+SR) | 0.67 ± 0.13 | 2.33 ± 0.41c | 3.33 ± 0.67c | 7.67 ± 1.84d |
| T3 (Tri3+SR) | 1.33 ± 0.31b | 1.33 ± 0.33de | 4.00 ± 0.52c | 9.33 ± 2.05c |
| T4 (Tri6+SR) | 2.00 ± 0.58b | 3.00 ± 0.45b | 7.00 ± 0.65b | 11.67 ± 1.87b |
| T5 (Tri2+ Tri3+ SR) | 0.00 ± 0.00e | 0.67 ± 0.10 | 1.00 ± 0.10e | 2.33 ± 0.11d |
| T6 (Tri2 + Tri6+ SR) | 0.33 ± 0.24d | 1.00 ± 0.14e | 1.33 ± 0.14e | 2.67 ± 0.15d |
| T7 (Tri3+ Tri6+ SR) | 1.00 ± 0.00c | 1.33 ± 0.19de | 2.00 ± 0.27d | 2.33 ± 0.17d |
| T8 (Tri2+Tri3+Tri6+ SR) | 0.33 ± 0.11d | 1.67 ± 0.15d | 0.93 ± 0.33e | 1.67 ± 0.34e |
| T9 (Provax-200+SR) | 0.00 ± 0.00e | 0.00 ± 0.00f | 0.00 ± 0.00f | 1.00 ± 0.18e |

*Note*: In the treatments, SR denotes inoculation with the Southern blight pathogen *Sclerotium rolfsii*, while Tri2, Tri3, and Tri6 represent treatments with *Trichoderma* isolates Tri2, Tri3, and Tri6, respectively. In T9, treatment with the fungicide Provax-200 was included*.* Values (mean ± SE) for each treatment were obtained from three biological replicates (*n = 3*). Different letters within each column indicate significant differences, as determined by Fisher’s LSD test (*p < 0.05*).
